# Human Milk Oligosaccharide Composition at 6 Weeks Is Associated with Temperament and Eating Behaviors of Children in the STRONG Kids 2 Cohort Through 4 Years of Age

**DOI:** 10.3390/nu17132080

**Published:** 2025-06-23

**Authors:** Yuting Fan, Kelly F. Bost, Sharon M. Donovan

**Affiliations:** 1 Department of Food Science and Human Nutrition, University of Illinois Urbana-Champaign, Urbana, IL 61801, USA; yutingf2@illinois.edu; 2 Human Development and Family Studies, Department of Human Development and Family Studies, University of Illinois Urbana-Champaign, Urbana, IL 61801, USA; kbost@illinois.edu; 3 Division of Nutritional Science, University of Illinois Urbana-Champaign, Urbana, IL 61801, USA

**Keywords:** human milk oligosaccharides, temperament, eating behaviors, behavioral development

## Abstract

**Background/Objectives:** Early childhood is a critical window of development, which is influenced by early life exposures including breastfeeding. Observational and preclinical studies have linked human milk oligosaccharides (HMOs) with neurocognitive development. However, less attention has focused on behavioral outcomes including temperament and eating behaviors. Herein, we investigated the associations between HMO consumption and child temperament and child eating behaviors through four years of age. **Methods**: Participants were drawn from the STRONG Kids 2 cohort. Human milk was collected at 6 weeks postpartum, and HMO relative abundances were determined by HPLC-MS. Using validated questionnaires, child temperament was assessed at 3, 18, and 48 months of age, and child eating behaviors were measured at 12, 18, 24, 36, and 48 months of age. After adjusting for covariates, multiple linear regressions were carried out to assess the relationship between HMOs and the outcome measures. **Results**: The HMO profile of mothers showed two distinct clusters explained by maternal secretor status. Significant associations between HMO and surgency were only found in the full cohort and secretors, while more associations between HMO and negative affectivity were observed in non-secretors. A significant number of associations was observed between HMOs and child eating behaviors in full cohort, secretors, and non-secretors. HMO diversity, total fucosylation, and total sialylation were positively associated with food responsiveness, while neutral HMOs presented negative associations. However, these associations with food responsiveness were not observed in non-secretors. **Conclusions**: HMO profiles were associated with behavioral outcomes in the children, with variations by child age and maternal secretor status, highlighting the potential role of HMOs within the broader context of maternal and postnatal influences.

## 1. Introduction

The first few years of life represent a critical and dynamic window for rapid growth and development [1], during which nutrition and environmental factors profoundly influence long-term cognitive [2] and behavioral development and health [3,4,5]. Human milk (HM) is the gold standard for infants by virtue of its short- and long-term health benefits [6]. Beyond meeting the nutritional needs of infants, HM plays an important role in immune protection [7], microbiome modulation [8], and cognitive development [9]. HM is a complex system that contains bioactive compounds, including 20–25 g/L of human milk oligosaccharides (HMOs) in colostrum and 5–20 g/L in mature HM [10,11]. HMOs are a structurally diverse group of carbohydrates that vary in composition among mothers [12]. The main compositional difference in HMO is driven by maternal genetics and secretor status, which is defined by the activity of the *FUT2* gene that encodes the α 1,2 fucosyltransferase enzyme. This enzyme enables the attachment of fucose residues to HMOs with an α 1–2 linkage, such as 2′-fucosyllactose (2′-FL), which is present in high concentrations in secretor mothers. In contrast, non-secretor mothers lack functional *FUT2* activity, resulting in a HMO profile with much lower or even absent levels of HMOs with α 1–2 linkages [12]. HMOs pass through the stomach and small intestine relatively intact until reaching the colon, where they exert their effects by modulating microbiota composition and function [13,14,15], reducing pathogen attachment to mucosal surfaces [16] and influencing systemic immunological responses [16,17].

Building on the beneficial roles of HMOs, they have been associated with infant neurocognitive development in recent years [18,19]. However, studies on the neurocognitive outcomes of HMO consumption in humans are underexplored, especially in terms of behavioral aspects. Also, no study to date has investigated associations between HMO composition and infant temperament or feeding behaviors. Temperament is considered a measure of personality [20]. Although there is no clear definition of the term, it is widely accepted as referring to the innate individual differences in responsiveness and self-regulation that emerge early in life, affect behavior, emotions, and attention, and remain relatively stable across the lifespan [21,22,23,24].

To assess the early life individual differences, three domains of temperament are usually measured: surgency, negative affectivity, and effortful control. Surgency, sometimes referred to as extraversion [25], is defined as an individual’s tendency toward positive emotions, sociability, and likelihood of engaging with others, seeking rewards, and enjoying high-energy activities [26]. Negative affectivity is the tendency to experience negative emotional states [27] and emotional stress, which can be associated with anxiety and depression [28]. Effortful control is the ability to inhibit a primary response in order to execute a secondary response [29] and reflects individual variations in the capacity to regulate emotions and behaviors in a self-directed manner [30]. Early life exposures are closely related to the later development of temperament [31,32].

Alongside temperament, early life eating behavior represents another crucial domain of child behavior that can be impacted by early environmental factors and can be linked to long-term health outcomes. The prevalence of childhood obesity has rapidly increased over the last few decades worldwide and has become a public health concern [33]. Obesity is a complex metabolic condition that can be attributed to various factors [34], including genetic predisposition, environmental impacts, dietary practices, family environment, lifestyles, socioeconomic status, etc. [35,36,37,38]. Among these, dietary patterns and eating behaviors play an important role in shaping weight status in childhood [39,40]. Eating behaviors involve the individual’s relationship with food and food consumption behaviors [41], which can include appetitive traits and food preferences [42]. Early eating behaviors establish a foundation for eating patterns and nutritional status later in life, which can be associated with eating disorders and the long-term risk of obesity [43]. Additionally, child temperament is significantly associated with eating behaviors [44,45]. Therefore, the primary objective of this study was to investigate relationships between early life exposure to HMO at 6 weeks postpartum and behavioral outcomes, including temperament and eating behaviors, through 4 years of age. We hypothesized that the relative abundances of sialylated and fucosylated HMOs at 6 weeks postpartum would be associated with infant and child temperament and eating behavior outcomes.

## 2. Materials and Methods

### 2.1. Participants and HMO Analysis

The participants are drawn from the STRONG Kids2 longitudinal birth cohort (IRB #13448) [46]. The STRONG Kids 2 cohort aims to identify early life determinants of growth and risk for childhood obesity. The participants were recruited from the central Illinois area in their third trimester of pregnancy (*n* = 468) [46]. From the entire cohort, 385 mothers who provided an HM sample at 6 weeks postpartum, and their breastfed babies were included in the current analysis. The method for HMO analysis using HPLC and the HMO profiles from the participants at 6 weeks postpartum were previously described [47]. Each HMO relative abundance was calculated as the proportion of each individual HMO relative to the total oligosaccharide content within each HM sample. The analysis included the relative abundances of the top 25 HMOs, HMO diversity, and the total relative abundance of the grouped sialylated, fucosylated, neutral, and non-fucosylated neutral HMOs. The participants reported breastfeeding exclusivity in online surveys at 1 week, 6 weeks, 3 months (mos), and 12 mos postpartum, which was used to estimate the breastfeeding duration. HMO relative abundances were normalized with the Gaussianization algorithm using the Lambert package in R (Version 4.2.1).

### 2.2. Covariates

Data were collected via online self-reported surveys [46]. Covariates include maternal age at delivery, pre-pregnancy BMI, maternal depression scores, maternal education level, and socioeconomic status at 6 weeks postpartum, infant birth weight, delivery mode, and breastfeeding duration. Maternal postnatal depression was measured by the Edinburgh Postnatal Depression Scale (EPDS) [48,49] at 6 weeks postpartum, which is the same time as the breast milk collection. The selection of covariates was based on data availability and previous literature to remove potential confounding.

### 2.3. Outcome Measures

When their child was 3 mos of age, the parent completed the Infant Behavior Questionnaire Revised-Very Short Form (IBQR-VS) [21,50], which measures infant temperament in three domains: surgency, negative affectivity, and effortful control. The effortful control component in infancy is labeled as “orienting/regulation” [51]. When the child was 18 and 48 mos old, the Early Childhood Behavior Questionnaire-Very Short (ECBQ-VS) [52,53] was completed to measure temperament in early childhood. The same three domains of temperament were assessed. The child’s early life eating behaviors were measured at 12 mos, 18 mos, and 2, 3, and 4 years old with the Children’s Eating Behaviour Questionnaire (CEBQ) [54]. This questionnaire has been validated across geographic locations around the world [55,56,57,58,59,60]. The CEBQ provides a practical measure of the child’s eating styles with a 35-item parent report questionnaire that generates eight scales, including “food approach” subscales (food responsiveness, enjoyment of food, emotion overeating, and desire to drink) and “food avoidant” subscales (food fussiness, slowness in eating, emotional undereating, and satiety responsiveness [54,55].

### 2.4. Statistical Analysis

All statistical analyses were performed in R (Version 4.2.1, R Foundation for Statistical Computing: Vienna, Austria, https://www.r-project.org/). Statistical significance was set at a *p*-value of ≤0.05. Multiple linear regression was used to assess the associations between HMO relative abundances and available outcome measures at each time point, including temperament as assessed by the IBQR-VS questionnaire and ECBQ-VS questionnaire, and child eating behaviors as assessed by the ECBQ questionnaire. A data matrix consisting of the top 25 HMOs was also analyzed with principal component analysis (PCA), a dimensionality reduction technique that transforms corrected variables into a smaller set of uncorrelated components, to identify patterns in HMO profiles in different mothers [61]. HMO relative abundances were log-transformed before analysis. Covariates were adjusted to control for potential confounding and were not independently tested as hypotheses [18]. All the categorical variables were standardized by one-hot encoding [62], and numerical variables were winsorized and standardized. Missing values were imputed before fitting into the models. Independence of residuals, homoscedasticity, and normality were not violated. Multiple testing was corrected using the Benjamini–Hochberg false discovery rate (FDR), where an adjusted *p*-value of ≤0.10 was considered significant. Given the exploratory nature of this study, we presented results both before and after applying FDR corrections.

## 3. Results

### 3.1. Summary Statistics for Demographic Information of the Participants

Descriptive statistics were computed for demographic information of the participants included in the current analysis. A summary of statistics is listed in Table 1.

### 3.2. HMO Clustering

**Figure 1:**
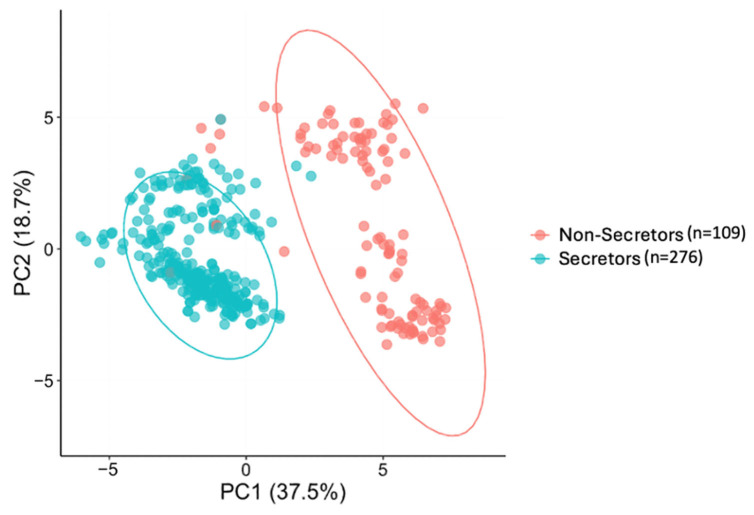
The variations of the top 25 human milk oligosaccharide (HMO) composition of mothers by secretor status using principal component analysis (PCA). Each point represents one maternal HMO profile. Secretor (blue circles, *n* = 276) and non-secretor (red circles, *n* = 109) groups are shown. The first two principal components (PC1 and PC2) are plotted, explaining 37.5% and 18.7% of the variance, respectively. The separation between the two circled groups reflects differences in overall HMO composition associated with the maternal secretor status.

A clear separation of samples was observed with PCA for the HMO composition of the mothers, suggesting inherent grouping within the dataset. The major differences between HMO profiles of the mothers are introduced by genetic variations or secretor status [63]. In our cohort, the secretor status explained 37.5% of the variation in HMO profiles (Figure 1). As previously reported, most of the top 25 most abundant HMO relative abundances varied by secretor status. Detailed information on the relative abundances of individual HMOs of secretor and non-secretor mothers was previously reported by Fan et. al. [18]. Therefore, in addition to analyzing the full cohort, associations between HMO relative abundances and the infant and child behavioral outcomes were examined within each secretor status group.

### 3.3. Associations Between HMO Profiles and Temperament Outcomes

Associations between HMO relative abundances and infant temperament were observed for all the sub-domains, with variations in the full cohort and by secretor status. Consistent with the terminology outlined in the Methods, we refer to the temperament domain of “effortful control” as “orienting/regulation” in infancy to reflect developmental changes in conceptualization. At 3 mos old, HMO diversity was found to be significantly associated with surgency (*β* = 0.128, *p* = 0.023), suggesting that higher HMO diversity is associated with increased surgency scores, but this was only observed in the full cohort. Individual neutral and fucosylated HMOs were also found to impact temperamental outcomes. Positive associations between 2′-FL and surgency scores were observed in both the full cohort and the secretor-only group. LNT+LNnT were negatively associated with infant surgency scores in the full cohort (*p* = 0.015) and in secretors (*p* = 0.036). Additionally, total nonfucosylated neutral HMOs (*β* = −0.127, *p* = 0.022) and LNFP I+LNFP III (*β* = −0.222, *p* = 0.021) are negatively associated with surgency scores in the full cohort. Conversely, non-secretors demonstrated unique positive associations between several fucosylated HMOs and infant negative affectivity, such as LNDFH I (*β* = 0.785, *p* = 0.010), and LNDFH 2 (*β* = 0.199, *p* = 0.029) (Table 2). However, after correcting for multiple testing, only the association between IFLNH III relative abundance and surgency scores at 3 mos remained statistically significant in the full cohort and secretors only (adjusted *p* = 0.08 and 0.04, respectively).

At 18 mos, the impact of HMOs on the surgency scores was less pronounced, and most effects were observed on effortful control and negative affectivity. However, at this point, the sialylated HMOs emerged as influencers for child temperamental outcomes. S-LNnH II appeared to be negatively associated with negative affectivity in the full cohort (*β* = −0.094, *p* = 0.009) and in non-secretors (*β* = −0.307, *p* = 0.002), suggesting that children of mothers with higher S-LNnH II relative abundances show lower negative affectivity. Total neutral HMO relative abundances were negatively associated with effortful control in the full cohort (*β* = −0.084, *p* = 0.040) and in secretors (*β* = −0.098, *p* = 0.045), but not in non-secretors. Furthermore, LNH appears to be negatively associated with negative affectivity at 18 mos old, which is consistent across the full cohort (*β* = −0.123, *p* = <0.001), secretors (*β* = −0.105, *p* = 0.040), and non-secretors (*β* = −0.150, *p* = 0.006). Interestingly, total sialylation was negatively associated with negative affectivity in non-secretors (*β* = −0.267, *p* = 0.002), indicating that sialylated HMOs, as a group, might be protective against negative affective behaviors. However, the finding diverged for one of the main sialylated HMOs, 3′-SL, which was positively associated with negative affectivity in non-secretors (*β* = 0.129, *p* = 0.034). The contrasting relationship suggests that the effects of sialylation on behavior are not uniform and could be influenced by the specific HMO (Table 3). After applying FDR correction, most of the associations at 18 mos of age were no longer significant in the full cohort and the secretors, except for the negative associations between LNH and negative affectivity in the full cohort (adjusted *p* = 0.02). Furthermore, a greater number of associations in non-secretors survived a 10% FDR correction, with total sialylation, S-LNnH II, and IFLNH III remaining negatively associated with negative affectivity (adjusted *p* = 0.1, 0.1, and 0.1, respectively).

We observed more associations between HMO relative abundances and temperamental outcomes in 4-year-old children. HMO structures containing lacto-N-hexaose (LNH) were consistently associated with negative affectivity in children, with notable differences by maternal secretor status. Specifically, in the full cohort, LNH, MFpLNH I+III, and DFLNHa all showed negative associations with negative affectivity. In secretors, MFpLNH I+III, and DFLNHa maintained their significant associations with negative affectivity in children. Similarly, in non-secretors, LNH, p-LNH, and MFpLNH I+III also exhibited negative associations with negative affectivity, further emphasizing the protective role of HMOs with similar structures. The only exception was noted for MFpLNH IV in non-secretors, which was positively associated with negative affectivity (Table 4). Additionally, FDR corrected results suggested that the negative associations between MFpLNH I+III and negative affectivity remained statistically significant in the full cohort (adjusted *p* = 0.009).

**Figure A1:**
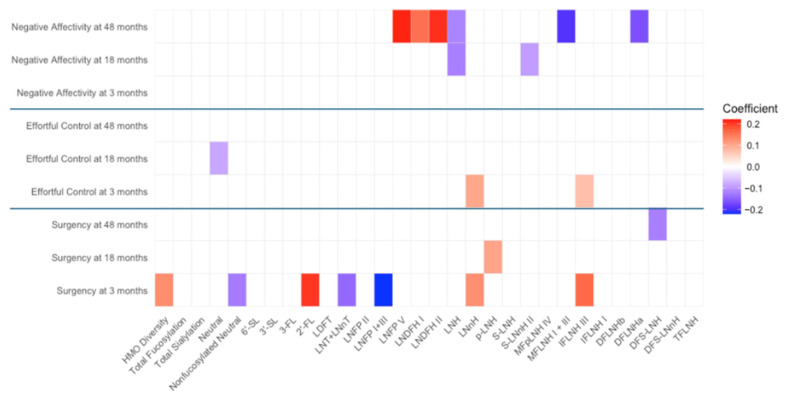
Heat map depicting the significant associations between HMO relative abundances at 6 weeks postpartum and child temperament sub-domain scores in full cohort (unadjusted). Multiple linear regressions were used after correcting for covariates. Associations that were statistically significant are shown in colors, with color intensity representing the strength and direction of the relationship. Non-significant associations are indicated in white-colored cells. Red colors indicate positive beta coefficients and purple colors indicate negative beta coefficient. HMO, human milk oligosaccharide; 2′-FL, 2′-fucosyllactose; 3-FL, 3-fucosyllactose; 3′-SL, 3′-sialyllactose; 6′-SL, 6′-sialyllactose; LNnT, lacto-N-neotetraose; LNT, lacto-N-tetrose; LDFT, lactodifucotetraose; LNFP I/II/III/V, lacto-N-fucopentaose-I/II/III/V; DFLNHa/b, difucosyllacto-N-hexaose a/b; LNDFH I/II, lacto-N-difucohexaose I/II; LNH, lacto-N-hexaose; LNnH, lacto-N-neohexaose; p-LNH, para-lacto-N-hexaose; S-LNH, sialyl-lacto-N-hexaose; S-LNnH II, sialyllacto-N-neohexaose II; MFpLNH IV, fucosyl-para-lacto-N-hexaose IV; MFLNH I/III, monofucosyllacto-N-hexaose I/III; IFLNH I/III, fucosyl-para-lacto-N-hexaose I/III; DFS-LNH, difucosylmonosialyllacto-N-hexaose; DFS-LNnH, difucosylmonosialyllacto-N-neohexaose; TFLNH, trifucosyllacto-N-hexaose.

**Figure A2:**
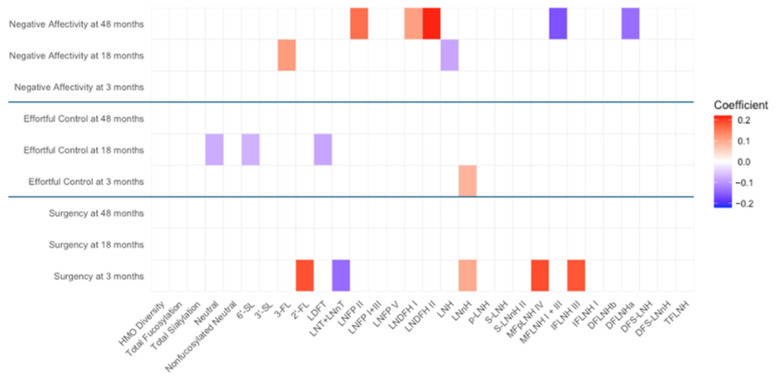
Heat map depicting the significant associations between HMO relative abundances at 6 weeks postpartum and child temperament sub-domain scores in secretors (unadjusted). Multiple linear regressions were used after correcting for covariates. Associations that were statistically significant are shown in colors, with color intensity representing the strength and direction of the relationship. Non-significant associations are indicated in white-colored cells. Red colors indicate positive beta coefficients and purple colors indicate negative beta coefficient. HMO, human milk oligosaccharide; 2′-FL, 2′-fucosyllactose; 3-FL, 3-fucosyllactose; 3′-SL, 3′-sialyllactose; 6′-SL, 6′-sialyllactose; LNnT, lacto-N-neotetraose; LNT, lacto-N-tetrose; LDFT, lactodifucotetraose; LNFP I/II/III/V, lacto-N-fucopentaose-I/II/III/V; DFLNHa/b, difucosyllacto-N-hexaose a/b; LNDFH I/II, lacto-N-difucohexaose I/II; LNH, lacto-N-hexaose; LNnH, lacto-N-neohexaose; p-LNH, para-lacto-N-hexaose; S-LNH, sialyl-lacto-N-hexaose; S-LNnH II, sialyllacto-N-neohexaose II; MFpLNH IV, fucosyl-para-lacto-N-hexaose IV; MFLNH I/III, monofucosyllacto-N-hexaose I/III; IFLNH I/III, fucosyl-para-lacto-N-hexaose I/ III; DFS-LNH, difucosylmonosialyllacto-N-hexaose; DFS-LNnH, difucosylmonosialyllacto-N-neohexaose; TFLNH, trifucosyllacto-N-hexaose.

**Figure A3:**
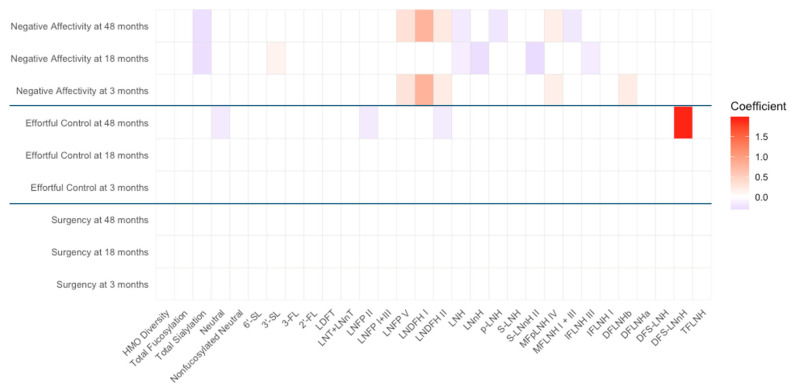
Heat map depicting the significant associations between HMO relative abundances at 6 weeks postpartum and child temperament sub-domain scores in non-secretors (unadjusted). Multiple linear regressions were used after correcting for covariates. Associations that were statistically significant are shown in colors, with color intensity representing the strength and direction of the relationship. Non -significant associations are indicated in white-colored cells. Red colors indicate positive beta coefficients and purple colors indicate negative beta coefficient. HMO, human milk oligosaccharide; 2′-FL, 2′-fucosyllactose; 3-FL, 3-fucosyllactose; 3′-SL, 3′-sialyllactose; 6′-SL, 6′-sialyllactose; LNnT, lacto-N-neotetraose; LNT, lacto-N-tetrose; LDFT, lactodifucotetraose; LNFP I/II/III/V, lacto-N-fucopentaose-I/II/III/V; DFLNHa/b, difucosyllacto-N-hexaose a/b; LNDFH I/II, lacto-N-difucohexaose I/II; LNH, lacto-N-hexaose; LNnH, lacto-N-neohexaose; p-LNH, para-lacto-N-hexaose; S-LNH, sialyl-lacto-N-hexaose; S-LNnH II, sialyllacto-N-neohexaose II; MFpLNH IV, fucosyl-para-lacto-N-hexaose IV; MFLNH I/III, monofucosyllacto-N-hexaose I/III; IFLNH I/III, fucosyl-para-lacto-N-hexaose I/III; DFS-LNH, difucosylmonosialyllacto-N-hexaose; DFS-LNnH, difucosylmonosialyllacto-N-neohexaose; TFLNH, trifucosyllacto-N-hexaose.

Overall, we observed that the associations between HMO and infant temperament varied based on maternal secretor status, with the effects on surgency being more pronounced in secretors and the effects on negative affectivity more pronounced in non-secretors (Figure A1, Figure A2 and Figure A3).

### 3.4. Associations Between HMO Profiles and Infant and Child Eating Behaviors

Eight subdomains of eating behaviors were investigated. Most scales were found to be associated with HMO composition at 6 weeks postpartum. In the full cohort at 12 mos of age, 6′-SL relative abundance was positively associated with enjoyment of food (*β* = 0.086, *p* = 0.028) and negatively associated with emotional undereating (*β* = −0.178, *p* < 0.001), suggesting the potential of 6′-SL to promote food and energy intake. In non-secretors, 6′-SL was also negatively associated with slowness in eating (*β* = −0.119, *p* = 0.032), further confirming the effect of 6′-SL to promote eating. Total sialylated HMO relative abundances were negatively associated with emotional undereating across the groups, which presents a consistent effect with 6′-SL. LNFP I + III relative abundances were negative predictors for enjoyment of food in the full cohort (*β* = −0.183, *p* = 0.018) and secretors (*β* = −0.201, *p* = 0.006), suggesting the potential to reduce food intake (Table 5). With FDR correction, the negative association between 6′-SL relative abundance and child emotional undereating behavior remained significant in the full cohort and in secretors (adjusted *p* = 0.09 and 0.014, respectively). In non-secretors, only the positive association between DFS-LNnH and emotional overeating continued to show significance (adjusted *p* = 0.08).

When the children were 18 mos old, the effects of 6′-SL on slowness in eating in the full cohort and secretors were still observed. Additionally, the relative abundance or total non-fucosylated neutral HMOs was a negative predictor for emotional overeating behavior (*β* = −0.066, *p* = 0.031) in the full cohort and food responsiveness in the secretors (*β* = −0.166, *p* = 0.002). LNDFH II was negatively associated with food fussiness in the full cohort (*β* = −0.141, *p* = 0.022) and in secretors (*β* = −0.278, *p* = 0.016). Total sialylation abundances were negatively associated with slowness in eating (*β* = −0.080, *p* = 0.040) and emotional undereating (*β* = −0.120, *p* = 0.029) in the full cohort and secretors (*β* = −0.088, *p* = 0.046 and *β* = −0.161, *p* = 0.008, respectively) (Table 6). However, the observed effects failed to withstand FDR correction.

When the children were 2 years old, some effects seen at earlier time points were still present. For instance, in the full cohort, total sialylation continued to be positively associated with enjoyment of food (*β* = 0.086, *p* = 0.045) and negatively associated with the “food avoidant” behaviors, including satiety responsiveness (*β* = −0.071, *p* = 0.038) and slowness in eating (*β* = −0.083, *p* = 0.037). Similar traits were also observed in secretors, but not in non-secretors. One of the most abundant sialylated HMOs, 6′-SL, displayed negative relationships with slowness in eating and emotional undereating in the full cohort and in secretors. Another sialylated HMO, S-LNH, was a positive predictor of emotional overeating and food responsiveness, and a negative predictor of satiety responsiveness and slowness in eating. These collectively suggest a role of S-LNH in promoting food intake, which is consistent with the relationship found for total sialylation. Total grouped abundances of the total non-fucosylated neutral HMOs were negatively associated with emotional overeating (*β* = −0.084, *p* = 0.015) and desire to drink (*β* = −0.121, *p* = 0.050) in the full cohort, suggesting their protective potential in unhealthy overeating behaviors (Table 7). However, after FDR corrections, the associations found at 24 mos were no longer statistically significant.

At 36 mos old, distinct effects of 3-FL between the secretor and the non-secretor groups were observed. Specifically, in the full cohort, 3-FL was positively associated with desire-to-drink behaviors and a tendency to approach sweetened beverages, which may contribute to higher caloric intake (*β* = 0.190, *p* = 0.012). This positive association was also significant within the secretor group (*β* = 0.231, *p* = 0.034). Conversely, in non-secretors, 3-FL demonstrated opposing effects. Higher relative abundances of 3-FL were negatively associated with enjoyment of food (*β* = −0.212, *p* = 0.007) and positively associated with satiety responsiveness (*β* = 0.203, *p* = < 0.001). Thus, depending on secretor status, 3-FL may play a role in modulating appetite and feeding behaviors differently (Table 8). It is important to note, however, that the findings fall below the threshold of significance after subsequent FDR correction.

The pattern of associations between HMOs and eating behaviors at 48 mos resembled that of 36-month-old children, suggesting more stabilized eating behaviors in early childhood. However, the effects of the total fucosylated HMO abundance on eating behaviors observed at 36 mos of age were no longer present (Table 9). Furthermore, the findings at 48 mos also failed to withstand FDR adjustment for multiple comparisons.

**Figure A4:**
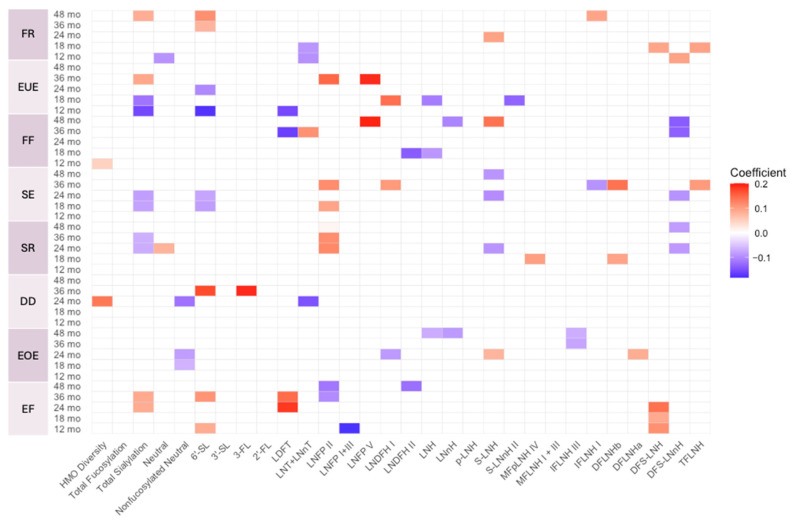
Heat map depicting the significant associations between HMO relative abundances at 6 weeks postpartum and child eating behavior sub-domain scores in full cohort (unadjusted). Multiple linear regressions were used after correcting for covariates. Associations that were statistically significant are shown in colors, with color intensity representing the strength and direction of the relationship. Non-significant associations are indicated in white-colored cells. Red colors indicate positive beta coefficients and purple colors indicate negative beta coefficient. Stars indicated statistical significance after FDR correction. HMO, human milk oligosaccharide; 2′-FL, 2′-fucosyllactose; 3-FL, 3-fucosyllactose; 3′-SL, 3′-sialyllactose; 6′-SL, 6′-sialyllactose; LNnT, lacto-N-neotetraose; LNT, lacto-N-tetrose; LDFT, lactodifucotetraose; LNFP I/II/III/V, lacto-N-fucopentaose-I/II/III/V; DFLNHa/b, difucosyllacto-N-hexaose a/b; LNDFH I/II, lacto-N-difucohexaose I/II; LNH, lacto-N-hexaose; LNnH, lacto-N-neohexaose; p-LNH, para-lacto-N-hexaose; S-LNH, sialyl-lacto-N-hexaose; S-LNnH II, sialyllacto-N-neohexaose II; MFpLNH IV, fucosyl-para-lacto-N-hexaose IV; MFLNH I/III, monofucosyllacto-N-hexaose I/III; IFLNH I/III, fucosyl-para-lacto-N-hexaose I/III; DFS-LNH, difucosylmonosialyllacto-N-hexaose; DFS-LNnH, difucosylmonosialyllacto-N-neohexaose; TFLNH, trifucosyllacto-N-hexaose; FR, food responsiveness; EUE, emotional undereating; FF, food fussiness; SE, slowness in eating; SR, satiety responsiveness; DD, desire to drink; EOE, emotional overeating.

**Figure A5:**
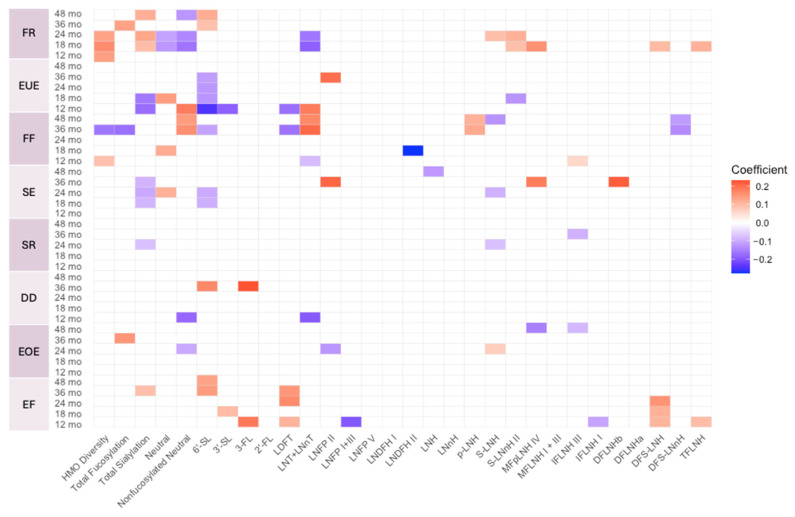
Heat map depicting the significant associations between HMO relative abundances at 6 weeks postpartum and child eating behavior sub-domain scores in secretors (unadjusted). Multiple linear regressions were used after correcting for covariates. Associations that were statistically significant are shown in colors, with color intensity representing the strength and direction of the relationship. Non-significant associations are indicated in white-colored cells. Red colors indicate positive beta coefficients and purple colors indicate negative beta coefficient. HMO, human milk oligosaccharide; 2′-FL, 2′-fucosyllactose; 3-FL, 3-fucosyllactose; 3′-SL, 3′-sialyllactose; 6′-SL, 6′-sialyllactose; LNnT, lacto-N-neotetraose; LNT, lacto-N-tetrose; LDFT, lactodifucotetraose; LNFP I/II/III/V, lacto-N-fucopentaose-I/II/III/V; DFLNHa/b, difucosyllacto-N-hexaose a/b; LNDFH I/II, lacto-N-difucohexaose I/II; LNH, lacto-N-hexaose; LNnH, lacto-N-neohexaose; p-LNH, para-lacto-N-hexaose; S-LNH, sialyl-lacto-N-hexaose; S-LNnH II, sialyllacto-N-neohexaose II; MFpLNH IV, fucosyl-para-lacto-N-hexaose IV; MFLNH I/III, monofucosyllacto-N-hexaose I/III; IFLNH I/III, fucosyl-para-lacto-N-hexaose I/III; DFS-LNH, difucosylmonosialyllacto-N-hexaose; DFS-LNnH, difucosylmonosialyllacto-N-neohexaose; TFLNH, trifucosyllacto-N-hexaose; FF, food fussiness; SE, slowness in eating; SR, satiety responsiveness; DD, desire to drink; EOE, emotional overeating.

**Figure A6:**
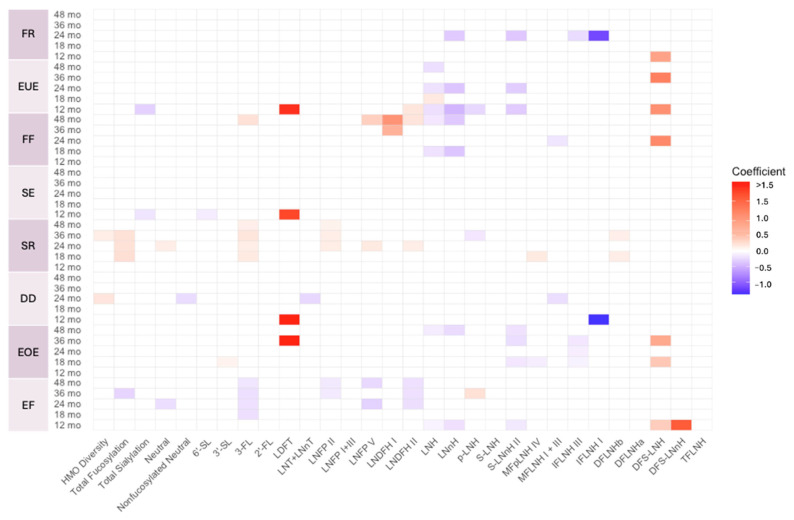
Heat map depicting the significant associations between HMO relative abundances at 6 weeks postpartum and child eating behavior sub-domain scores in non-secretors (unadjusted). Multiple linear regressions were used after correcting for covariates. Associations that were statistically significant are shown in colors, with color intensity representing the strength and direction of the relationship. Non-significant associations are indicated in white-colored cells. Red colors indicate positive beta coefficients and purple colors indicate negative beta coefficient. HMO, human milk oligosaccharide; 2′-FL, 2′-fucosyllactose; 3-FL, 3-fucosyllactose; 3′-SL, 3′-sialyllactose; 6′-SL, 6′-sialyllactose; LNnT, lacto-N-neotetraose; LNT, lacto-N-tetrose; LDFT, lactodifucotetraose; LNFP I/II/III/V, lacto-N-fucopentaose-I/II/III/V; DFLNHa/b, difucosyllacto-N-hexaose a/b; LNDFH I/II, lacto-N-difucohexaose I/II; LNH, lacto-N-hexaose; LNnH, lacto-N-neohexaose; p-LNH, para-lacto-N-hexaose; S-LNH, sialyl-lacto-N-hexaose; S-LNnH II, sialyllacto-N-neohexaose II; MFpLNH IV, fucosyl-para-lacto-N-hexaose IV; MFLNH I/III, monofucosyllacto-N-hexaose I/III; IFLNH I/III, fucosyl-para-lacto-N-hexaose I/III; DFS-LNH, difucosylmonosialyllacto-N-hexaose; DFS-LNnH, difucosylmonosialyllacto-N-neohexaose; TFLNH, trifucosyllacto-N-hexaose; FF, food fussiness; SE, slowness in eating; SR, satiety responsiveness; DD, desire to drink; EOE, emotional overeating.

In summary, many associations were identified between HMO profiles and child eating behavior sub-domains. Some distinct patterns were seen in secretors and non-secretors. For example, most effects of HMO on food responsiveness were observed in secretors, while most effects on satiety responsiveness were found in non-secretors (Figure A4, Figure A5 and Figure A6). However, most of the associations were no longer significant after correcting for multiple testing (Table 10). Only several associations remained to be associated with emotional eating behaviors at 12 mos.

## 4. Discussion

Herein, associations between HMO composition and infant temperament and eating behaviors were investigated in the STRONG Kids 2 cohort. HMOs have been associated with various aspects of neurocognitive development in intervention trials in preclinical animal models, as well as in association studies in human infants [18,19]. However, the effects of HMO on the development of temperament traits and eating behaviors are underexplored. While our initial analyses revealed a great number of associations between HMO profile and infant behavioral outcomes, many did not remain statistically significant following FDR corrections for individual HMO and behavior sub-domains. However, given the lack of extant data, the unadjusted findings can also help inform future studies in this area.

Temperament development in early childhood is vital to how children respond to and interact with the environment around them [64] and predicts later personality and behaviors [65], including psychopathology [66]. We first investigated and found associations between maternal HMO compositions at 6 weeks postpartum and child temperamental traits (surgency, effortful control, and negative affectivity), with variations among maternal secretor status and child ages. During infancy, 2′-FL and LNnH positively affected surgency and effortful control (orienting/regulation) in the full cohort and infants of mothers who are secretors, but these associations did not persist through childhood. In the total cohort, positive relationships between HMO diversity and infant surgency scores were only observed at 3 mos. Additionally, common associations between similarly structured HMO relative abundances and temperamental outcomes were found, but some differences in directionality of these relationships were noted.

Sialylated HMOs have been associated with neurocognitive development [67] and anxiety reduction [68]. The sialic acid component has been suggested to up-regulate the mRNA levels in the hippocampus and liver for sialic acid biosynthesis. Sialic acid is an essential part of brain gangliosides that modulate dopamine receptor function [69], influencing early childhood temperament development [70,71]. Therefore, we investigated the effects of sialylated HMOs on infant and child temperamental outcomes. Indeed, total sialylated HMOs were negatively associated with negative affectivity in non-secretors at 18 and 48 mos old, which supported the mood-regulating effects of sialic acid. Furthermore, the sialylated HMO, S-LNnH II, was also negatively associated with negative affectivity in the full cohort and in non-secretors when the children were 18 mos old. However, 3′-SL showed an opposite effect in non-secretors at 18 mos old. Likewise, several LNH-containing HMOs, including LNH, MFpLNH I+III, and DFLNHa, consistently showed negative associations with negative affectivity across the full cohort, secretors, and non-secretors. In the full cohort, negative associations between LNH and negative affectivity at 18 mos and negative associations between MFLNH I+III and negative affectivity at 48 mos survived 10% FDR correction, further suggesting the similar effects of LNH-containing HMOs. The exception was MFpLNH IV, which exhibited positive associations with negative affectivity, mirroring the variability we observed for 3′-SL and suggesting the complexity of HMO-behavioral interactions.

Given that HMOs have been observed to exhibit potential effects on temperament, it is crucial to further investigate this early life nutritional exposure on other behavioral aspects, including eating patterns. CEBQ is a validated measurement for child eating behaviors that involves eight sub-scales. Those sub-scales have been extensively reported to be an important predictor for the later development of obesity. Food responsiveness and enjoyment of food are positive predictors of increased weight-for-length z scores in infancy, and slowness in eating is a negative predictor [72,73]. Satiety responsiveness was reported to be a negative predictor for child BMI z scores [74]. In the STRONG Kids 2 cohort, we identified associations between HMO relative abundances and eating behavior sub-scales.

Plows et al. explored the associations between HMO composition and child eating behaviors, and this was the only other study that considered child eating behavior as an outcome measurement [75]. Their study utilized the Baby Eating Behaviour Questionnaire (BEBQ), an adaptation of the CEBQ for younger babies. Eating behaviors were assessed at 1 and 6 mos of age via the parent report survey. The authors reported that LNnT was negatively associated with food responsiveness in the total sample [75]. In our cohort, Negative associations were also observed between LNT+LNnT relative abundance in the full cohort at 12 mos, at 18 mos, and in secretors. Plows et al. also reported that at 6 mos, FLNH, LNH, and DSLNH were positively associated with food responsiveness in both the total sample and secretors only [75]. Similarly, in our cohort, we discovered positive associations between HMO structures with the LNH component and food responsiveness subdomain at younger ages close to the sampling times of the authors, although these results did not survive the FDR corrections. For example, at 12 mos of age, DFS-LNH and TFLNH were positively associated with food responsiveness in the full cohort and secretors; S-LNnH II and MFpLNH IV were positively associated with food responsiveness in secretors only. At 24 mos old, S-LNH was positively associated with food responsiveness in the full cohort and secretors. These results provided additional evidence of the potential positive associations between LNH residue and overeating behaviors.

Children of mothers with pre-pregnancy overweight and obesity have been reported to at higher risk for childhood obesity [76], and it has been suggested that feeding practices may serve as the mechanism for intergeneration transmission of obesity [76,77]. To contextualize our current findings, we revisited our study on maternal determinants of HMO composition. Our previous analysis in this cohort showed that 3-FL and LNFP V were negatively associated with maternal pre-pregnancy BMI in non-secretor mothers [47]. In the current analysis, we observed that when the children are 18 mos old, 3-FL relative abundances were negative predictors for enjoyment of food and positive predictors for satiety responsiveness. These associations suggest that higher 3-FL abundances may play a protective role against overeating behaviors in children, a relationship that persists consistently at 24, 36, and 48 mos, but this was only seen in non-secretors. At 24 mos old, LNFP V also appeared to be negatively associated with enjoyment of food and positively associated with satiety responsiveness. Furthermore, we previously demonstrated that higher LNT and LNnT relative abundance is associated with lower maternal obesity [47], and we found that greater LNT and LNnT abundance is associated with lower food responsiveness behavior in children at 12 and 18 mos old. These eating behaviors could potentially, in turn, reduce the risk of developing childhood obesity. Furthermore, it has been suggested that maternal BMI is a positive predictor for the concentration of 6′-SL [78]. In the present study, the association for eating behaviors that remained significant after FDR correction is the negative association between 6‘-SL relative abundance and emotional undereating behavior when the child is 1 year old (adjusted *p*-value = 0.014), suggesting an opposite effect of 6′-SL on food avoidance actions. Thus, we can hypothesize that maternal pre-pregnancy BMI could indirectly impact the child’s risk of obesity in childhood via HMO composition and subsequent behavioral outcomes, warranting further investigation.

Additionally, in the STRONG Kids 2 cohort, we investigated the impacts of pregnancy complications, including Pregnancy-Induced Hypertension and Gestational Diabetes Mellitus (GDM). We discovered that mothers who developed GDM showed higher relative abundances of individual HMOs, including LDFT, LNDFH I, LNnH, S-LNnH II, and DFS-LNH [47]. In the current study, some of these HMOs were also associated with child eating behaviors at 2 years of age. For instance, in the total cohort and among secretors, LDFT and DFS-LNH were positively associated with enjoyment of food, whereas LNnH and S-LNnH II were negatively associated with emotional undereating behaviors. Interestingly, LNDFH I was found to be protective against the emotional overeating behavior. Although these findings highlight both connections and discrepancies, it is still worth noting the potential of maternal-infant interactions on child development through the modulation of HMO composition in breastmilk, emphasizing the need for further investigation in this area. It has been reported that there is a link between maternal GDM and offspring overweight and obesity [79,80]. This increased risk of childhood obesity in children born to mothers with GDM reflects the fetal programming [81,82]. Maternal metabolic conditions like obesity or inflammatory conditions, including GDM, might alter the bioactive components such as HMO composition in breastmilk [83,84]

Surprisingly, although we identified a protective potential of 3-FL in excessive eating behaviors in non-secretors, a different relationship was observed in the full cohort and in secretors. This may suggest that 3-FL influences behaviors through distinct pathways based on maternal genetic background and secretor status. Such findings highlight the importance of considering individual variations when examining the effects of HMO on child development. It has previously been reported that the milk microbiome can vary largely by maternal secretor status [63]. It has also been suggested that the extent of HMO impact on child developmental outcomes also depends on maternal secretor status [85].

Across the time points for the eating behavior assessment, we observed associations between LNFP II relative abundances and “food avoidant” behaviors such as satiety responsiveness, suggesting the potential of LNFP II to protect from childhood obesity. This is in agreement with Berger et al. [86], who reported that higher LNFP II predicts lower weight-for-age z scores at 6 mos. Although this relationship is more pronounced in older ages after 18 mos and did not survive FDR corrections, the potential of LNFP II for preventing rapid weight gain in early life is still worth noting.

Overall, we defined variations in the effects of HMO on temperament and eating behaviors, which could potentially be attributed to the interaction of HMO with gut microbiome, the complexity of HMO metabolites, and the gut–brain axis. The gut–brain axis is the bidirectional interaction between the gut and the brain, which regulates physiological functions including mood, immune functions, and food intake [87,88]. Several infant gut bacterial species can metabolize HMOs and produce metabolites, including short-chain fatty acids (SCFAs) [89]. SCFAs can increase the expression of blood–brain barrier tight junctions and then reduce the permeability [90]. Appetite and energy intake can also be regulated via SCFA-stimulated hormonal and neuronal signals [91]. Additionally, SCFAs such as butyrate and acetate can upregulate the serotonin pathway, and serotonin is a neurotransmitter that regulates the mood and appetite [92]. Therefore, HMO can potentially exert effects on child temperament and eating behaviors via the regulation of SCFA production.

We also found that the effects of HMO on eating behaviors were quite consistent between 3 and 4 years old, which further supports the hypothesis that the effects of HMO were achieved via the modulation of the gut microbiome, as the gut microbiome composition is likely to become relatively stable beyond 3 years of age [93]. The links between gut microbiome environment and eating behaviors via the gut–brain axis have been well studied in adults [94,95,96]. However, studies on younger children in this area are relatively limited. Berding et al. also mentioned in their analysis of the associations between gut microbiome composition and dietary patterns that the differences in the children’s dietary patterns were associated with unique microbiome compositions. A dietary pattern that contains refined carbohydrates, sweetened beverages, snacks, and sweets was linked to a higher relative abundance of *Bacteroidetes*, *Bacteroides*, and *Ruminococcus*, along with a lower abundance of *Bifidobacterium*, *Prevotella*, *Blautia*, and *Roseburia* [97]. The microbiome patterns are likely influenced by the HMO composition and, then, influence child feeding patterns in turn. For example, with our knowledge of the ability of *Bifidobacterium longum* subsp. *infantis* to utilize HMOs, including 3-FL [98], the positive associations observed between satiety response and 3-FL relative abundances might be partially explained by the promotion of *Bifidobacterium* growth. Thus, it will be valuable to further investigate the mediating role of gut microbiome in the relationships between HMO profile and child eating behaviors, which is currently under investigation in our cohort.

Additionally, child temperament can influence eating behavior development [99] and childhood obesity risk [100]. For example, children with a temperament style marked by greater negative affectivity were at greater risk of developing unhealthful eating patterns [101], supporting the results observed in the STRONG Kids2 cohort at 4 years of age. Lower negative affectivity was observed in children of non-secretors who had consumed HMO with higher LNH relative abundances. At the same time, lower emotional overeating scores were also found in children who consumed a greater abundance of LNH, suggesting the potential protective role of LNH in unhealthy eating behavior development and childhood obesity.

While our findings suggest associations between HMO exposure at 6 weeks postpartum and child behavioral outcomes, we were unable to interpret those relationships as causal. The composition of HMOs is determined by a range of complex factors, including genetics, epigenetic regulation, environmental factors, dietary habits, and health status. These maternal factors may also affect child development via additional biological interactions. Furthermore, HMOs might exert effects on child development via indirect pathways such as the gut–brain axis. Future research integrating multi-omics data will be needed to disentangle the complex interactions.

### Strengths and Limitations

A strength of the current study was the ability to obtain infant and child behavioral outcomes at multiple time points up to 4 years of age with a high participant retention rate, allowing us to explore the impact of early life exposures over a critical development window. Additionally, the focus of the study on the impact of HMO composition on behavioral traits provides a unique perspective on the effects of early life nutrition on child development trajectories.

This study also has some limitations. First, our study population has a relatively high level of education and socioeconomic status, which may not fully represent the broader population. Also, HMO profiles were reported as a relative abundance of the total HMO composition rather than the absolute concentration, which did not allow estimation of actual amounts of individual HMO intake by the infants. Moreover, we only had HM samples available at one time point and were not able to quantify the changes in HMO concentrations across all stages of lactation. Lastly, given the limited number of prior studies in this area, the current analysis was conducted in an exploratory framework. Separate regression analyses were performed for each time point and subscale measures for behavioral outcomes with the purpose of providing an initial understanding of the potential relationships between HMO exposure and infant behavioral development. After the implementation of a 10% FDR correction for multiple testing, fewer associations remained significant. Thus, the results of this analysis need to be considered as tentative and interpreted for hypothesis generation for further studies.

## 5. Conclusions

This study provides insights into the associations between the HMO composition at 6 weeks postpartum and subsequent infant and child temperament and eating behavior outcomes. Our findings suggest that individual HMOs and grouped HMOs relative abundances, as well as HMO diversity, may shape early behavioral traits and feeding patterns. However, the effect of HMOs varies by maternal secretor status and child age. The traits of temperament and eating behaviors are important in the early developmental period, as they can set the foundations for lifelong dietary habits and can play a role in the development of obesity and metabolic disorders.

## Figures and Tables

**Table 1:** Summary statistics of demographic information in the STRONG Kids 2 cohort.

Characteristic	*n* (%) or Mean ± SD
Maternal age (yrs)	31.2 ± 4.2
Pre-pregnancy BMI	26.3 ± 6.2
Maternal depression EPDS score (on a scale of 0–30)	4.3 ± 4.0
Infant birthweight (g)	3492.7 ± 428.4
Infant sex assigned at birth	
Female	179 (46.5%)
Male	206 (53.5%)
Secretor status	
Secretor	276 (71.7%)
Non-secretor	109 (28.3%)
Delivery mode	
Cesarean section	88 (22.9%)
Vaginal	294 (76.4%)
Missing	3 (0.8%)
Maternal education level at enrollment	
Grade school/some high school/high school graduate	13 (3.4%)
Some college or technical school	56 (14.5%)
College graduate/post-graduate work	303 (78.7%)
Missing	13 (3.4%)
Socioeconomic status (monthly income)	
USD 3000 and under	97 (25.2%)
USD 3001–5000	104 (27.0%)
USD 5001 and above	136 (35.3%)
Missing	48 (12.5%)
Maternal Ethnicity	
Hispanic/Latino	15 (3.9%)
Non-Hispanic/Latino Unknown or Mixed	61 (15.8%)
Non-Hispanic/Latino White	285 (74.0%)
Missing	24 (6.2%)
Child Ethnicity	
Hispanic/Latino	19 (4.9%)
Non-Hispanic/Latino Unknown or Mixed	74 (19.2%)
Non-Hispanic/Latino White	270 (70.1%)
Missing	22 (5.7%)
Exclusive Breastfeeding duration	
6 weeks	24 (6.2%)
3 mos	107 (27.8%)
12 mos	197 (51.2%)
Mixed feeding (human milk and formula)	49 (12.7%)
Missing	8 (2.1%)

Abbreviations: mos, months; yrs, years; SD, standard deviation; BMI, body mass index; EPDS, Edinburgh Postnatal Depression Scale.

**Table 2:** Associations between HMO and temperament at three months of age (unadjusted for false discovery).

Sample	Temperament Characteristic	HMO	Estimate	*p*-Values
Full cohort	Surgency	HMO Diversity	0.128	0.023
		Total non-fucosylated neutral	−0.127	0.022
		2′-FL	0.204	0.028
		LNT+LNnT	−0.145	0.015
		LNFP I+LNFP III	−0.222	0.021
		LNnH	0.127	0.025
		IFLNH III	0.164	<0.001
	Orienting/ Regulation	LNnH	0.101	0.014
		IFLNH III	0.073	0.039
Secretors	Surgency	2′-FL	0.229	0.015
		LNT+LNnT	−0.175	0.036
		LNnH	0.123	0.046
		MFpLNH IV	0.234	0.024
		IFLNH III	0.224	<0.001
	Orienting/ Regulation	LNnH	0.108	0.014
Non-Secretors	Negative affectivity	LNFP V	0.288	0.016
		LNDFH I	0.785	0.010
		LNDFH II	0.199	0.029
		MFpLNH IV	0.168	0.046
		DFLNHb	0.209	0.028

Abbreviations: 2′-FL, 2′-fucosyllactose; LNnT, lacto-N-neotetraose; LNT, lacto-N-tetrose; LNFP I/III/V, lacto-N-fucopentaose-I/III/V; DFLNHb, difucosyllacto-N-hexaose b; LNDFH I/II, lacto-N-difucohexaose I/II; LNnH, lacto-N-neohexaose; MFpLNH IV, fucosyl-para-lacto-N-hexaose IV; IFLNH III, fucosyl-para-lacto-N-hexaose III.

**Table 3:** Associations between HMO and temperament at 18 months of age (unadjusted for false discovery).

Sample	Temperament Characteristic	HMO	Estimate	*p*-Values
Full Cohort	Surgency	p-LNH	0.104	0.012
	Effortful control	Neutral	−0.084	0.040
	Negative affectivity	LNH	−0.123	<0.001
		S-LNnH II	−0.094	0.009
Secretors	Effortful control	Neutral	−0.098	0.045
		6′-SL	−0.092	0.038
		LDFT	−0.105	0.040
	Negative affectivity	3-FL	0.142	0.018
		LNH	−0.105	0.014
Non-Secretors	Negative affectivity	Total Sialylation	−0.267	0.002
		3′-SL	0.129	0.034
		LNH	−0.150	0.006
		LNnH	−0.284	0.019
		S-LNnH II	−0.307	0.002
		IFLNH III	−0.159	0.003

Abbreviations: 3-FL, 3-fucosyllactose; 3′-SL, 3′-sialyllactose; 6′-SL, 6′-sialyllactose; LDFT, lactodifucotetraose; LNH, lacto-N-hexaose; LNnH, lacto-N-neohexaose; p-LNH, para-lacto-N-hexaose; S-LNnH II, sialyllacto-N-neohexaose; IFLNH III, fucosyl-para-lacto-N-hexaose III.

**Table 4:** Associations between HMO and temperament at 48 months of age (unadjusted for false discovery).

Sample	Temperament Characteristic	HMO	Estimate	*p*-Values
Full cohort	Surgency	DFS-LNH	−0.123	0.026
	Negative affectivity	LNFP V	0.220	0.008
		LNDFH I	0.157	0.007
		LNDFH II	0.208	0.002
		LNH	−0.118	0.008
		MFpLNH I+III	−0.198	<0.001
		DFLNHa	−0.168	0.004
Secretors	Negative affectivity	LNFP II	0.197	0.025
		LNDFH I	0.136	0.022
		LNDFH II	0.275	0.030
		MFpLNH I+III	−0.206	0.004
		DFLNHa	−0.173	0.003
Non-Secretors	Effortful control	Neutral	−0.179	0.033
		LNFP II	−0.180	0.012
		LNDFH II	−0.172	0.047
		DFS-LNnH	1.988	0.050
	Negative affectivity	Total Sialylation	−0.280	0.020
		LNFP V	0.309	0.012
		LNDFH I	0.767	0.011
		LNDFH II	0.231	0.012
		LNH	−0.176	0.019
		p-LNH	−0.212	0.044
		MFpLNH IV	0.174	0.048
		MFpLNH I+III	−0.190	0.018

Abbreviations: LNFP II/V, lacto-N-fucopentaose-II/V; DFLNHa, difucosyllacto-N-hexaose a; LNDFH I/II, lacto-N-difucohexaose I/II; LNH, lacto-N-hexaose; p-LNH, para-lacto-N-hexaose; MFpLNH IV, fucosyl-para-lacto-N-hexaose IV; DFS-LNH, difucosylmonosialyllacto-N-hexaose; DFS-LNnH, difucosylmonosialyllacto-N-neohexaose.

**Table 5:** Associations between HMO and eating behaviors at 12 months of age (unadjusted for false discovery).

Sample	Eating Behavior	HMO	Estimate	*p*-Values
Full Cohort	Enjoyment of food	6′-SL	0.086	0.028
		LNFP I+III	−0.183	0.018
		DFS-LNH	0.114	0.019
	Food fussiness	HMO Diversity	0.048	0.038
	Emotional undereating	Total Sialylation	−0.158	0.004
		6′-SL	−0.178	<0.001
		LDFT	−0.156	0.032
	Food responsiveness	Neutral	−0.094	0.025
		LNT+LNnT	−0.096	0.043
		DFS-LNnH	0.096	0.033
Secretors	Enjoyment of food	3-FL	0.186	0.008
		LDFT	0.110	0.047
		LNFP I+III	−0.201	0.006
		IFLNH I	−0.109	0.022
		DFS-LNH	0.108	0.021
		TFLNH	0.095	0.047
	Desire to drink	Total nonfucosylated neutral	−0.180	0.021
		LNT+LNnT	−0.198	0.025
	Food fussiness	HMO Diversity	0.088	0.002
		LNT+LNnT	−0.080	0.011
		IFLNH III	0.058	0.016
	Emotional undereating	Total Sialylation	−0.176	0.005
		Total nonfucosylated neutral	0.182	0.020
		6′-SL	−0.250	<0.001
		3′-SL	−0.190	0.003
		LDFT	−0.172	0.023
		LNT+LNnT	0.183	0.039
	Food responsiveness	HMO Diversity	0.138	0.021
Non-Secretors	Emotional overeating	LNH	−0.079	0.016
		LNnH	−0.183	0.016
		S-LNnH II	−0.142	0.015
		DFS-LNH	0.396	0.026
		DFS-LNnH	1.482	<0.001
	Slowness in eating	Total Sialylation	−0.172	0.029
		6′-SL	−0.119	0.032
		LDFT	4.36	0.031
	Desire to drink	LDFT	7.47	0.022
		IFLNH I	−1.383	0.014
	Emotional undereating	Total Sialylation	−0.291	0.017
		LDFT	6.24	0.046
		LNDFH II	0.191	0.028
		LNH	−0.202	0.006
		LNnH	−0.473	0.006
		p-LNH	−0.248	0.016
		S-LNnH II	−0.335	0.011
		DFS-LNH	0.851	0.033
	Food responsiveness	DFS-LNH	0.711	0.036

Abbreviations: 3-FL, 3-fucosyllactose; 3′-SL, 3′-sialyllactose; 6′-SL, 6′-sialyllactose; LNnT, lacto-N-neotetraose; LNT, lacto-N-tetrose; LDFT, lactodifucotetraose; LNFP I/III, lacto-N-fucopentaose-I/III; LNDFH II, lacto-N-difucohexaose II; LNH, lacto-N-hexaose; LNnH, lacto-N-neohexaose; p-LNH, para-lacto-N-hexaose; S-LNnH II, sialyllacto-N-neohexaose II; IFLNH I/III, fucosyl-para-lacto-N-hexaose I/ III; DFS-LNH, difucosylmonosialyllacto-N-hexaose; DFS-LNnH, difucosylmonosialyllacto-N-neohexaose; TFLNH, trifucosyllacto-N-hexaose.

**Table 6:** Associations between HMO and eating behaviors at 18 months (unadjusted for false discovery).

Sample	Eating Behavior	HMO	Estimate	*p*-Values
Full Cohort	Enjoyment of food	DFS-LNH	0.089	0.050
	Emotional overeating	Total Nonfucosylated neutral	−0.066	0.031
	Satiety responsiveness	MFpLNH IV	0.099	0.022
		DFLNHb	0.094	0.045
	Slowness in eating	Total Sialylation	−0.080	0.040
		6′-SL	−0.084	0.017
		LNFP II	0.094	0.035
	Food fussiness	LNDFH II	−0.141	0.022
		LNH	−0.090	0.030
	Emotional undereating	Total Sialylation	−0.120	0.029
		LNDFH I	0.142	0.039
		LNH	−0.112	0.029
		S-LNnH II	−0.134	0.017
	Food responsiveness	LNT+LNnT	−0.091	0.050
		DFS-LNH	0.093	0.042
		TFLNH	0.097	0.041
Secretors	Enjoyment of food	3′-SL	0.100	0.028
		DFS-LNH	0.114	0.011
	Slowness in eating	Total Sialylation	−0.088	0.046
		6′-SL	−0.097	0.027
	Emotional undereating	Total Sialylation	−0.161	0.008
		Neutral	0.142	0.041
		6′-SL	−0.128	0.034
		S-LNnH II	−0.130	0.035
	Food fussiness	Neutral	0.120	0.029
		LNDFH II	−0.278	0.016
	Food responsiveness	HMO Diversity	0.163	0.005
		Total Sialylation	0.095	0.030
		Neutral	−0.125	0.012
		Total nonfucosylated neutral	−0.166	0.002
		LNT+LNnT	−0.191	0.002
		S-LNnH II	0.091	0.040
		MFpLNH IV	0.157	0.049
		DFS-LNH	0.100	0.025
		TFLNH	0.113	0.014
Non-Secretors	Enjoyment of food	3-FL	−0.203	0.014
	Emotional overeating	3′-SL	0.092	0.035
		S-LNnH II	−0.151	0.038
		MFpLNH IV	−0.117	0.012
		IFLNH III	−0.091	0.028
		DFS-LNH	0.434	0.044
	Satiety responsiveness	Total Fucosylation	0.261	0.005
		3-FL	0.168	0.013
		MFpLNH IV	0.165	0.007
		DFLNHb	0.142	0.032
	Emotional undereating	LNH	−0.171	0.045
	Food fussiness	LNH	−0.186	0.005
		LNnH	−0.364	0.014

Abbreviations: 3-FL, 3-fucosyllactose; 3′-SL, 3′-sialyllactose; 6′-SL, 6′-sialyllactose; LNnT, lacto-N-neotetraose; LNT, lacto-N-tetrose; LNFP II, lacto-N-fucopentaose-II; DFLNHb, difucosyllacto-N-hexaose b; LNDFH I/II, lacto-N-difucohexaose I/II; LNH, lacto-N-hexaose; LNnH, lacto-N-neohexaose; S-LNnH II, sialyllacto-N-neohexaose II; MFpLNH IV, fucosyl-para-lacto-N-hexaose IV; IFLNH III, fucosyl-para-lacto-N-hexaose III; DFS-LNH, difucosylmonosialyllacto-N-hexaose; TFLNH, trifucosyllacto-N-hexaose.

**Table 7:** Associations between HMO and eating behaviors at 24 months (unadjusted for false discovery).

Sample	Eating Behavior	HMO	Estimate	*p*-Values
Full cohort	Enjoyment of food	Total Sialylation	0.086	0.045
		LDFT	0.183	0.001
		DFS-LNH	0.140	0.003
	Emotional overeating	Total Nonfucosylated neutral	−0.084	0.015
		LNDFH I	−0.088	0.035
		S-LNH	0.078	0.018
		DFLNHa	0.085	0.044
	Desire to drink	HMO Diversity	0.134	0.031
		Total Nonfucosylated neutral	−0.121	0.050
		LNT+LNnT	−0.150	0.023
	Satiety responsiveness	Total Sialylation	−0.071	0.038
		Neutral	0.079	0.017
		LNFP II	0.120	0.002
		S-LNH	−0.093	0.007
		DFS-LNnH	−0.089	0.012
	Slowness in eating	Total Sialylation	−0.083	0.037
		6′-SL	−0.077	0.032
		S-LNH	−0.099	0.014
		DFS-LNnH	−0.094	0.025
	Emotional undereating	6′-SL	−0.103	0.030
	Food responsiveness	S-LNH	0.096	0.024
Secretors	Enjoyment of food	LDFT	0.163	0.004
		DFS-LNH	0.153	0.001
	Emotional overeating	Total nonfucosylated neutral	−0.101	0.018
		LNFP II	−0.126	0.036
		S-LNH	0.072	0.026
	Satiety responsiveness	Total Sialylation	−0.076	0.043
		S-LNH	−0.076	0.028
	Slowness in eating	Total Sialylation	−0.104	0.020
		Neutral	0.113	0.023
		6′-SL	−0.100	0.022
		S-LNH	−0.094	0.022
	Emotional undereating	6′-SL	−0.125	0.030
	Food responsiveness	HMO Diversity	0.132	0.024
		Total Sialylation	0.127	0.005
		Neutral	−0.110	0.027
		Total nonfucosylated neutral	−0.144	0.009
		LNT+LNnT	−0.164	0.008
		S-LNH	0.094	0.022
		S-LNnH II	0.114	0.012
Non-Secretors	Enjoyment of food	Neutral	−0.214	0.011
		3-FL	−0.190	0.026
		LNFP V	−0.303	0.009
		LNDFH II	−0.195	0.021
	Emotional overeating	IFLNH III	−0.116	0.034
	Desire to drink	HMO Diversity	0.211	0.035
		Total nonfucosylated neutral	−0.229	0.026
		LNT+LNnT	−0.254	0.017
		MFLNH I+III	−0.216	0.019
	Satiety responsiveness	Total Fucosylation	0.231	0.010
		Neutral	0.133	0.033
		3-FL	0.144	0.022
		LNFP II	0.149	0.003
		LNFP V	0.169	0.048
		LNDFH II	0.135	0.031
	Emotional undereating	LNH	−0.185	0.021
		LNnH	−0.368	0.040
		S-LNnH II	−0.321	0.031
	Food fussiness	MFLNH I+III	−0.167	0.027
		DFS-LNH	0.905	0.021
	Food responsiveness	LNnH	−0.340	0.041
		S-LNnH II	−0.349	0.011
		IFLNH III	−0.231	0.002
		IFLNH I	−1.186	0.024

Abbreviations: 3-FL, 3-fucosyllactose; 6′-SL, 6′-sialyllactose; LNnT, lacto-N-neotetraose; LNT, lacto-N-tetrose; LDFT, lactodifucotetraose; LNFP II/V, lacto-N-fucopentaose-II/V; DFLNHa, difucosyllacto-N-hexaose a; LNDFH I/II, lacto-N-difucohexaose I/II; LNH, lacto-N-hexaose; LNnH, lacto-N-neohexaose; S-LNH, sialyl-lacto-N-hexaose; S-LNnH II, sialyllacto-N-neohexaose II; MFLNH I/III, monofucosyllacto-N-hexaose I/III; IFLNH I/III, fucosyl-para-lacto-N-hexaose I/III; DFS-LNH, difucosylmonosialyllacto-N-hexaose; DFS-LNnH, difucosylmonosialyllacto-N-neohexaose.

**Table 8:** Associations between HMOs and eating behaviors at 36 months (unadjusted for false discovery).

Sample	Eating Behavior	HMO	Estimate	*p*-Values
Full Cohort	Enjoyment of Food	Total Sialylation	0.089	0.037
		6′-SL	0.109	0.004
		LDFT	0.145	0.009
		LNFP II	−0.101	0.036
	Emotional overeating	IFLNH III	−0.077	0.028
	Desire to drink	6′-SL	0.170	0.002
		3-FL	0.190	0.012
	Satiety responsiveness	Total Sialylation	−0.069	0.046
		LNFP II	0.116	0.003
	Slowness in eating	LNFP II	0.116	0.014
		LNDFH I	0.104	0.047
		IFLNH I	−0.094	0.049
		DFLNHb	0.139	0.015
		TFLNH	0.104	0.033
	Food fussiness	LDFT	−0.164	0.006
		LNT+LNnT	0.113	0.032
		DFS-LNnH	−0.137	0.004
	Emotional undereating	Total Sialylation	−0.108	0.045
		LNFP II	0.149	0.014
		LNFP V	0.189	0.049
	Food responsiveness	6′-SL	0.078	0.048
Secretors	Enjoyment of food	Total Sialylation	0.091	0.049
		6′-SL	0.142	0.002
		LDFT	0.147	0.007
	Emotional overeating	Total fucosylation	0.150	0.015
	Desire to drink	6′-SL	0.168	0.011
		3-FL	0.231	0.034
	Satiety responsiveness	IFLNH III	−0.093	0.022
	Slowness in eating	Total Sialylation	−0.090	0.046
		LNFP II	0.210	0.006
		MFpLNH IV	0.183	0.024
		DFLNHb	0.219	0.014
	Food fussiness	HMO Diversity	−0.163	0.015
		Total Fucosylation	−0.173	0.027
		Total nonfucosylated neutral	0.159	0.012
		6′-SL	−0.110	0.031
		LDFT	−0.170	0.005
		LNT+LNnT	0.205	0.004
		p-LNH	0.122	0.020
		DFS-LNnH	−0.139	0.003
	Emotional undereating	6′-SL	−0.119	0.038
		LNFP II	0.198	0.046
	Food responsiveness	Total Fucosylation	0.134	0.049
		6′-SL	0.089	0.043
Non-Secretors	Enjoyment of food	Total Fucosylation	−0.270	0.022
		3-FL	−0.212	0.007
		LNFP II	−0.136	0.039
		LNDFH II	−0.193	0.016
		p-LNH	0.237	0.005
	Emotional overeating	LDFT	6.897	0.019
		S-LNnH II	−0.219	0.024
		IFLNH III	−0.155	0.014
		DFS-LNH	0.664	0.045
	Satiety responsiveness	HMO Diversity	0.140	0.017
		Total Fucosylation	0.293	0.001
		3-FL	0.203	<0.001
		LNFP II	0.135	0.007
		p-LNH	−0.150	0.024
		DFLNHb	0.129	0.050
	Emotional undereating	DFS-LNH	0.960	0.046
	Food fussiness	LNDFH I	0.606	0.028

Abbreviations: 3-FL, 3-fucosyllactose; 6′-SL, 6′-sialyllactose; LNnT, lacto-N-neotetraose; LNT, lacto-N-tetrose; LDFT, lactodifucotetraose; LNFP II/V, lacto-N-fucopentaose-II/V; DFLNHb, difucosyllacto-N-hexaose b; LNDFH I/II, lacto-N-difucohexaose I/II; p-LNH, para-lacto-N-hexaose; S-LNnH II, sialyllacto-N-neohexaose II; MFpLNH IV, fucosyl-para-lacto-N-hexaose IV; IFLNH I/III, fucosyl-para-lacto-N-hexaose I/III; DFS-LNH, difucosylmonosialyllacto-N-hexaose; DFS-LNnH, difucosylmonosialyllacto-N-neohexaose; TFLNH, trifucosyllacto-N-hexaose.

**Table 9:** Associations between HMOs and eating behaviors at 48 months (unadjusted for false discovery).

Sample	Eating Behavior	HMO	Estimate	*p*-Values
Full Cohort	Enjoyment of Food	LNFP II	−0.119	0.014
		LNDFH II	−0.125	0.035
	Emotional overeating	LNH	−0.071	0.046
		LNnH	−0.088	0.024
		IFLNH III	−0.070	0.037
	Satiety responsiveness	LNFP II	0.010	0.017
		DFS-LNnH	−0.085	0.023
	Slowness in eating	S-LNH	−0.092	0.041
	Food fussiness	LNFP V	0.200	0.019
		LNnH	−0.106	0.038
		S-LNH	0.139	0.004
		DFS-LNnH	−0.141	0.005
	Food responsiveness	Total Sialylation	0.084	0.040
		6′-SL	0.114	0.003
		IFLNH I	0.094	0.047
Secretors	Enjoyment of food	6′-SL	0.132	0.008
	Emotional overeating	MFpLNH IV	−0.153	0.042
		IFLNH III	−0.085	0.047
	Slowness in eating	LNH	−0.123	0.037
	Food fussiness	Total nonfucosylated neutral	0.143	0.029
		LNT+LNnT	0.166	0.023
		p-LNH	0.112	0.041
		S-LNH	−0.131	0.006
		DFS-LNnH	−0.121	0.012
	Food responsiveness	Total Sialylation	0.116	0.010
		Total nonfucosylated neutral	−0.126	0.026
		6′-SL	0.120	0.010
Non-Secretors	Enjoyment of food	3-FL	−0.165	0.032
		LNFP II	−0.143	0.026
		LNFP V	−0.246	0.016
		LNDFH II	−0.192	0.012
	Emotional overeating	LNH	−0.119	0.024
		LNnH	−0.236	0.020
		S-LNnH II	−0.176	0.036
	Satiety response	3-FL	0.125	0.039
		LNFP II	0.104	0.041
	Emotional undereating	LNH	−0.210	0.022
	Food fussiness	3-FL	0.234	0.014
		LNFP V	0.367	0.003
		LNDFH I	0.828	0.009
		LNDFH II	0.222	0.021
		LNH	−0.155	0.050
		LNnH	−0.344	0.023

Abbreviations: 3-FL, 3-fucosyllactose; 6′-SL, 6′-sialyllactose; LNnT, lacto-N-neotetraose; LNT, lacto-N-tetrose; LNFP II/V, lacto-N-fucopentaose-II/V; LNDFH I/II, lacto-N-difucohexaose I/II; LNH, lacto-N-hexaose; LNnH, lacto-N-neohexaose; S-LNH, sialyl-lacto-N-hexaose; S-LNnH II, sialyllacto-N-neohexaose II; MFpLNH IV, fucosyl-para-lacto-N-hexaose IV; IFLNH III, fucosyl-para-lacto-N-hexaose  III; DFS-LNnH, difucosylmonosialyllacto-N-neohexaose.

**Table 10:** Statistically significant associations between HMOs and infant behaviors after applying FDR correction for multiple testing.

Sample	Temperament Characteristics and Eating Behaviors	HMO	Estimate	FDR Adjusted *p*-Values
Full Cohort	Surgency (3 mo)	IFLNH III	0.164	0.08
	Negative affectivity (18 mo)	LNH	−0.123	0.02
	Negative affectivity (48 mo)	MFLNH I+III	−0.206	0.009
	Emotional undereating (12 mo)	6′-SL	−0.178	0.09
Secretors	Surgency (3 mo)	IFLNH III	0.224	0.024
	Emotional undereating (12 mo)	6′-SL	−0.25	0.014
Non-Secretors	Negative affectivity (18 mo)	Total Sialylation	−0.267	0.10
	Negative affectivity (18 mo)	S-LNnH II	−0.307	0.10
	Negative affectivity (18 mo)	IFLNH III	−0.159	0.10
	Emotional overeating (12 mo)	DFS-LNnH	1.482	0.08

Abbreviations: mo, month; 6′-SL, 6′-sialyllactose; IFLNH III, fucosyl-para-lacto-N-hexaose III; LNH, lacto-N-hexaose; MFpLNH I+III, fucosyl-para-lacto-N-hexaose I+III; S-LNnH II, sialyllacto-N-neohexaose II; DFS-LNnH, difucosylmonosialyllacto-N-neohexaose.

## Data Availability

The data presented in this study are available on request from the corresponding author due to privacy reasons.

## Abbreviations

The following abbreviations are used in this manuscript:

2′-FL	2′-Fucosyllactose
3-FL	3-Fucosyllactose
3′-SL	3′-Sialyllactose
6′-SL	6′-Sialyllactose
LNnT	Lacto-N-neotetraose
LNT	Lacto-N-tetrose
LDFT	Lactodifucotetraose
LNFP I/II/III/V	Lacto-N-fucopentaose-I/II/III/V
DFLNHa/b	Difucosyllacto-N-hexaose a/b
LNDFH I/II	Lacto-N-difucohexaose I/II
LNH	Lacto-N-hexaose
LNnH	Lacto-N-neohexaose
p-LNH	Para-lacto-N-hexaose
S-LNH	Sialyl-lacto-N-hexaose
S-LNnH II	Sialyllacto-N-neohexaose II
MFpLNH IV	Fucosyl-para-lacto-N-hexaose IV
MFLNH I + III	Monofucosyllacto-N-hexaose I/III
IFLNH I/III	Fucosyl-para-lacto-N-hexaose I/III
DFS-LNH	Difucosylmonosialyllacto-N-hexaose
DFS-LNnH	Difucosylmonosialyllacto-N-neohexaose
TFLNH	Trifucosyllacto-N-hexaose
HMO	Human Milk Oligosaccharides
HPLC-MS	High performance liquid chromatography-mass spectrometry
BMI	Body Mass Index
EPDS	Edinburgh Postnatal Depression Scale
IBQR-VS	Infant Behavior Questionnaire Revised-Very Short
ECBQ-VS	Early Childhood Behavior Questionnaire-Very Short
CEBQ	Children’s Eating Behaviour Questionnaire
BEBQ	Baby Eating Behaviour Questionnaire
yr(s)	Year/Years
mo(s)	Month/Months
FR	Food responsiveness
EUE	Emotional undereating
FF	Food fussiness
SE	Slowness in eating
SR	Satiety responsiveness;
DD	Desire to drink
EOE	Emotional overeating
GDM	Gestational diabetes mellitus
SCFAs	Short-chain fatty acids
